# Puumala Virus RNA in Patient with Multiorgan Failure

**DOI:** 10.3201/eid1202.050634

**Published:** 2006-02

**Authors:** Stefan Hoier, Stephan W. Aberle, Cord Langner, Wolfgang Schnedl, Christoph Högenauer, Emil C. Reisinger, Günter J. Krejs, Robert Krause

**Affiliations:** * Medical University of Graz, Graz, Austria;; † Medical University of Vienna, Vienna, Austria;; ‡ University of Rostock, Rostock, Germany

**Keywords:** puumala, bone marrow, multiorgan failure

**To the Editor:** The hantaviruses (genus *Hantavirus*, family *Bunyaviridae*) include human pathogens and occur worldwide (*1*). In Western and Central Europe, the predominant serotype is Puumala virus (PUUV), which causes epidemic nephropathy. We report the first Austrian patient with reverse transcription–polymerase chain reaction (RT-PCR)–confirmed PUUV infection and, to our knowledge, the first detection of PUUV-specific RNA in bone marrow.

On April 27, 2004, a previously healthy 52-year-old bus driver stopped his bus because of visual disturbance, dizziness, headache, and weakness in his legs; he then lost consciousness for a few minutes. He was seen at the neurology emergency service and subsequently admitted to the university hospital in Graz. He smoked tobacco, drank beer on the weekends, and cleaned his bus in the garage daily. The patient showed slight paresis of the right leg, nystagmus, cognitive deficit, and retrograde amnesia. Laboratory tests showed increases in (normal values are shown in parentheses) C-reactive protein (CRP) 40 mg/L (<9), creatine kinase (CK) 224 U/L (<170), lactate dehydrogenase (LDH) 244 U/L (<240), and myoglobin 416 ng/mL (<90). Cerebrospinal fluid showed elevated protein of 60 mg/dL (<45) but no other abnormalities. Results of computed tomographic scan of the brain and chest radiograph were normal. Because of increasing CRP (115 mg/L), empiric antimicrobial therapy with piperacillin/tazobactam was started. During an electroencephalogram on April 29, the patient deteriorated and was admitted to the intensive care unit for respiratory failure with a partial oxygen pressure of 40 mm Hg; he required intubation and mechanical ventilation. A chest radiograph showed diffuse pulmonary infiltration and slight bilateral pleural effusion. Laboratory examination showed CRP 265 mg/L, CK 42,570 U/L, LDH 1,235 U/L, myoglobin >3,000 ng/mL, aspartate aminotransferase 368 U/L (<35), alanine aminotransferase 96 U/L (<45), γ-glutamyl transpeptidase 182 U/L (<55), erythrocytes 3.76 × 10^9^/mL, leukocytes 9.09 × 10^6^/mL, thrombocytopenia of 9.2 × 10^4^ platelets/mL, and lymphoplasmacytoid cells on peripheral blood smear. Serum electrophoresis and immunofixation showed an increased γ-globulin fraction with oligoclonal immunoglobulin G (IgG) λ and IgGκ components. A bone marrow biopsy showed hypercellularity and 15% lymphoid cells with plasmocytoid features. Fluorescence-activated cell sorter testing showed 3% reactive B- and T-cell blasts but no signs of a malignant hematologic disease. Culture of bronchoalveolar lavage for bacteria and fungi was negative. Urinary antigen tests for *Legionella* spp. and pneumococci were negative. Serum antibody tests for *Leptospira* spp. were negative, but IgM against PUUV was detected by POC Puumala rapid test (Erilab Ltd, Kuopio, Finland) and recomLine Bunyavirus IgG/IgM test (Mikrogen, Martinsried, Germany). PUUV RNA was detectable in serum and in bone marrow by RT-PCR (*2*). PUUV was confirmed with a bootstrap probability of 99% on phylogenetic analysis (*2*). On May 1, status epilepticus developed and was treated with clonazepam. On May 2, renal function deteriorated and progressed to a maximum serum creatinine concentration of 4 mg/dL (0.6–1.3) and urea of 244 mg/dL (10–45), which required hemodialysis. CRP increased to 360 mg/L, and blood pressure decreased to 95/65 mmHg. The patient received intensive supportive care including dopamine and norepinephrine. After improvement, the patient was extubated on May 9. Eight days later, fever (temperature up to 40°C), *Enterococcus faecalis* bacteremia, nosocomial pneumonia from methicillin-resistant *Staphylococcus aureus*, respiratory failure requiring mechanic ventilation, and renal failure developed in the patient. Despite antimicrobial drug therapy with linezolid, the patient died 19 days after reintubation.

In Austria, before this case, PUUV RNA had only been detected by RT-PCR in rodents (*2*). We report the first Austrian patient with RT-PCR–confirmed PUUV infection. Furthermore, PUUV-specific RNA had never been detected in bone marrow. In animal studies, PUUV induces production of proinflammatory cytokines, such as interleukin (IL)-6 and IL-10 (*3*). IL-6 constitutes a major growth factor for myeloma and plasma cells, induces immunoglobulin production, and is an active factor in B-cell differentiation (*4**,**5*). IL-10 is a differentiation factor for plasma cell formation and immunoglobulin secretion. Since we detected a clear increase of IL-6, IL-10, and tumor necrosis factor α (TNFα) during the acute phase of infection (IL-6 133.0 pg/dL, IL-10 218.0 U/mL, and TNFα 29.7 pg/mL), we assume that lymphoplasmacytoid cells in bone marrow and peripheral blood of our patient and his production of oligoclonal γ-globulins were due to PUUV-induced cytokine release. Epidemic nephropathy usually takes a benign course, but multiorgan failure with cerebral involvement developed in our patient. Whereas neurologic symptoms such as headache (97% of patients), blurred vision (40%), and vomiting (31%) are common in patients infected with PUUV, only a few cases have been reported with severe central nervous system involvement (i.e., meningitis, epileptiform seizures) (*6**,**7*). Our patient had visual disturbances, slight paresis of the right leg, nystagmus, cognitive deficit, retrograde amnesia, and status epilepticus. We want to draw attention to the severe course PUUV infections can rarely take. The presence of PUUV in bone marrow explains the marked hematologic changes with lymphoplasmacytoid cells in marrow and peripheral blood.

## References

[1] Lee HW. Epidemiology and pathogenesis of haemorrhagic fever with renal syndrome. In: Eliott RM, editor. The *Bunyaviridae*. New York: Plenum Press; 1996. p. 253–67.

[2] Aberle SW, Lehner P, Ecker M, Aberle JH, Arneitz K, Khanakah G, Nephropathia epidemica and Puumala virus in Austria. Eur J Clin Microbiol Infect Dis. 1999;18:467–72. 10.1007/s100960050325 10482022

[3] Klingstroem J, Plyusnin A, Vaheri A, Lundkvist A. Wild-type Puumala hantavirus infection induces cytokines, C-reactive protein, creatinine, and nitric oxide in cynomolgus macaques. J Virol. 2002;76:444–9. 10.1128/JVI.76.1.444-449.2002 11739712 PMC135710

[4] Burdin N, Van Kooten C, Galibert L, Abrams JS, Wijdenes J, Banchereau J, Endogenous IL-6 and IL-10 contribute to the differentiation of CD40-activated human B lymphocytes. J Immunol. 1995;154:2533–44. 7533177

[5] Rousset F, Garcia E, Defrance T, Peronne C, Vezzio N, Hsu DH, Interleukin 10 is a potent growth and differentiation factor for activated human B lymphocytes. Proc Natl Acad Sci U S A. 1992;89:1890–3. 10.1073/pnas.89.5.1890 1371884 PMC48559

[6] Alexeyev OA, Morozov VG. Neurological manifestations of hemorrhagic fever with renal syndrome caused by Puumala virus: review of 811 cases. Clin Infect Dis. 1995;20:255–8. 10.1093/clinids/20.2.255 7742425

[7] Krause R, Aberle SW, Haberl R, Daxboeck F, Wenisch C. Puumala virus infection with acute disseminated encephalomyelitis and multiorgan failure. Emerg Infect Dis. 2003;9:603–5. 12737748 10.3201/eid0905.020405 PMC2972754

